# Slender Sheath/Guiding Catheter Combination vs. Sheathless Guiding Catheter for Acute Coronary Syndrome: A Propensity-Matched Analysis of the Two Devices

**DOI:** 10.1155/2020/8216831

**Published:** 2020-08-14

**Authors:** Tsuyoshi Isawa, Kazunori Horie, Taku Honda, Masataka Taguri, Norio Tada

**Affiliations:** ^1^Department of Cardiology, Sendai Kousei Hospital, Sendai, Japan; ^2^Department of Data Science, Yokohama City University School of Data Science, Yokohama, Japan

## Abstract

A Glidesheath slender (Terumo, Tokyo, Japan) and a sheathless Eaucath guiding catheter (Asahi Intecc, Nagoya, Japan) are two major slender devices utilized in percutaneous coronary intervention (PCI). This study aimed to investigate the differences in access-site complications between these devices in PCI for acute coronary syndrome (ACS). A total of 1108 consecutive patients who underwent transradial PCI for ACS were enrolled. Transradial PCI was performed using either a 7-Fr Glidesheath slender/7-Fr guiding catheter combination (Glidesheath group) or a 7.5-Fr sheathless guiding catheter (Sheathless group); 1 : 1 propensity score matching was performed, and 728 patients (364 in each group) were included in the propensity-matched population. In the matched patients, univariate analysis revealed that the Glidesheath group had less radial artery occlusion (RAO) at 30 days (Glidesheath: 1.4% vs. Sheathless: 4.1%, odds ratio (OR) = 0.33, 95% confidence interval (CI) =  0.12–0.91, *p*=0.039), whereas no significant between-group differences were observed in severe radial spasm (Glidesheath: 1.4% vs. Sheathless: 1.9%, OR = 0.71, 95% CI = 0.23–2.22, *p*=0.58) or access-site major bleeding (Glidesheath: 1.4% vs. Sheathless: 1.6%, OR = 0.83, 95% CI = 0.26–2.71, *p*=1.00). Multivariate analysis revealed that the choice for Glidesheath was significantly associated with less RAO (OR = 0.32, 95% CI = 0.11–0.93, *p*=0.036). In conclusion, 7-Fr Glidesheath slender/7-Fr guiding catheter combination is obviously more advantageous than 7.5-Fr sheathless guiding catheters for decreased risk of RAO. The potential low risk of RAO in our findings supports the adoption of the 7-Fr Glidesheath slender sheath/7-Fr guiding catheter combination in transradial PCI for ACS.

## 1. Introduction

**Figure 1:**
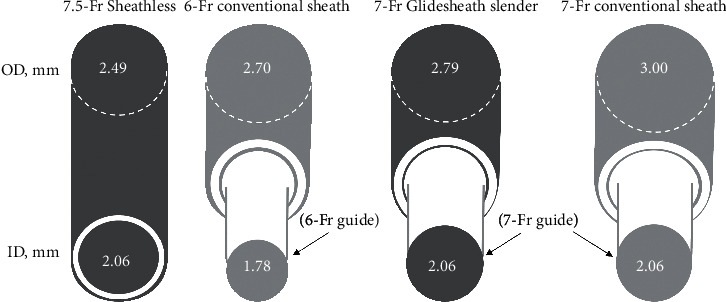
Comparison of the outer diameter and inner lumen diameters among the Glidesheath slender sheath, sheathless guiding catheter, and conventional sheath (illustration prepared by the authors with reference to the Glidesheath slender® sheath and Sheathless Eaucath® information brochures). “Glidesheath slender” denotes a Glidesheath slender sheath, and “Sheathless” denotes a sheathless guiding catheter. ID, inner lumen diameter; OD, outer diameter.

The standard treatment for acute coronary syndrome (ACS) is transradial percutaneous coronary intervention (PCI), which has considerably better outcomes than transfemoral PCI [1]. Although the default approach for PCI is via radial access, access-site complications, like radial artery occlusion (RAO) [2] and radial spasm [3], are still encountered due to overstretch and injury of the radial artery. Several slender (downsized) devices have been developed based on the hypothesis that smaller guiding catheters or sheaths reduce the risk of access-site complications. Two such devices are Glidesheath slender sheath (Terumo, Tokyo, Japan), which is a thin-walled radial sheath, and a sheathless Eaucath guiding catheter (Asahi Intecc, Nagoya, Japan), which is a guiding catheter system without a conventional sheath [4–6]. Regarding the device profile, difference in the outer diameter between a 7-Fr Glidesheath slender and 7.5-Fr sheathless guiding catheter is 0.30 mm (Figure 1). The outer diameters of the 7.5-Fr sheathless guiding catheter and the 7-Fr Glidesheath slender are smaller than, or almost equal to, that of the conventional 6-Fr sheath, which is currently used as the mainstream sheath in transradial PCI. During preparation for PCI for ACS, it is reasonable to select either a 7-Fr Glidesheath slender system or 7.5-Fr sheathless guiding system. They provide better backup support than the conventional 6-Fr system, and they are sufficiently downsized and consequently may lead to less or equal radial overstretch and injury than the conventional 6-Fr system. The rates of RAO associated with 7-Fr Glidesheath slender sheaths or 7.5-Fr sheathless guiding catheters are expected to be lower than, or almost equal to, those of the 6-Fr conventional sheath. Furthermore, they are better than the conventional 6-Fr system for optimal aspiration of thrombus. Larger caliber devices can aspirate a larger volume of thrombus as a result of the increased proximal cross-sectional area of the aspiration device. Although routine thrombus aspiration is not recommended, in cases of a large residual thrombus burden after opening the vessel with a guidewire or a balloon, thrombus aspiration is still considered [7]. Previous reports have demonstrated access-site complications in transradial PCI using Glidesheath slender sheaths (RAO 0.8%–4.8% and radial spasm 2.3%–11.0%) and using sheathless guiding catheters (RAO 0.0%–3.2% and radial spasm 0.7%–5.0%) [5, 6, 8–10]. However, no previous study has compared the 7-Fr Glidesheath slender/7-Fr guiding catheter combination with the 7.5-Fr sheathless guiding catheter in terms of the access-site complications in a large sample of patients with ACS treated with transradial PCI. Therefore, we aimed to investigate the differences between the 7-Fr Glidesheath slender sheath/7-Fr guiding catheter combination and 7.5-Fr sheathless Eaucath guiding system in terms of access-site complications in transradial PCI for ACS, using propensity score matching analysis.

## 2. Materials and Methods

### 2.1. Patient Selection

**Figure 2:**
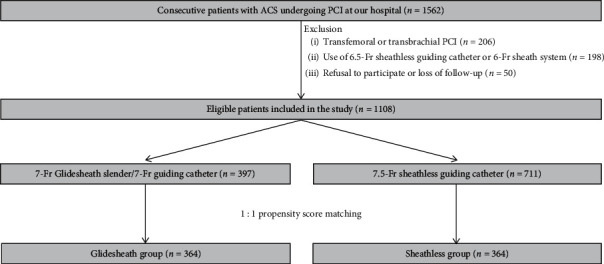
Flowchart of study design and patient selection criteria. ACS, acute coronary syndrome; PCI, percutaneous coronary intervention.

This was a single-center retrospective study that included patients with ACS who underwent transradial PCI in which either the 7-Fr Glidesheath slender/7-Fr guiding catheter or 7.5-Fr sheathless guiding catheter was used.  Figure 2 shows a flowchart of the study design and patient selection criteria. We screened 1562 consecutive patients who underwent PCI for ACS, including ST-elevation myocardial infarction and non-ST-elevation ACS, at the Sendai Kousei Hospital between January 2015 and February 2019. The exclusion criteria were as follows: (i) PCI via femoral or brachial access, (ii) transradial PCI using guiding catheter systems other than 7.5-Fr sheathless Eaucath guiding catheters or 7-Fr Glidesheath slender sheaths/7-Fr Hyperion guiding catheter (Asahi Intecc) combination, (iii) refusal to participate, and (iv) loss of follow-up. Consequently, we retrospectively evaluated 1108 patients who underwent transradial PCI using 7-Fr Glidesheath slender sheath/7-Fr Hyperion guiding catheter combination (Glidesheath group) (*n* = 397) or 7.5-Fr sheathless Eaucath guiding catheter (Sheathless group) (*n* = 711).

### 2.2. PCI Procedure and Radial Ultrasound Study

**Figure 3:**
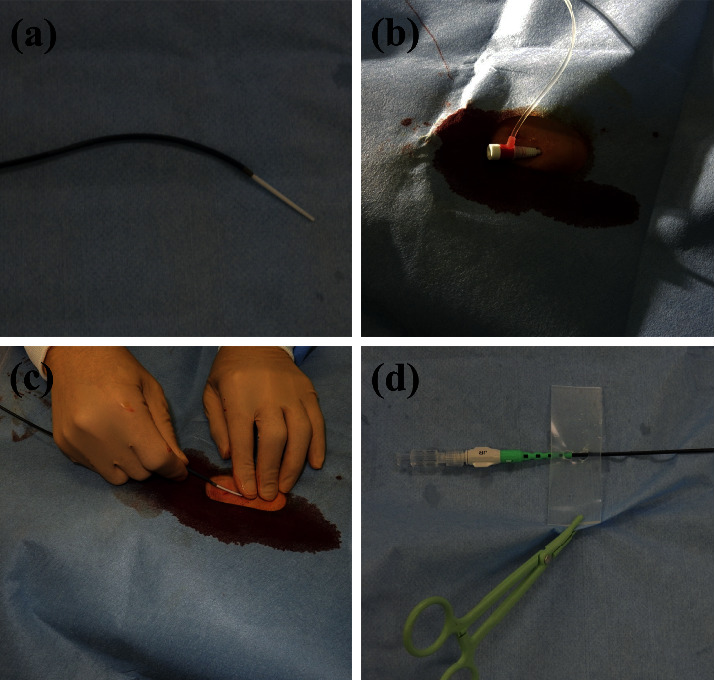
Sheathless transradial percutaneous coronary intervention procedure. (a) A central dilator is introduced into a guiding catheter. (b) A 4-Fr conventional sheath is inserted into the radial artery. (c) Following introduction, the sheath is exchanged for a sheathless guiding catheter connected to a supplied central dilator over a 0.035-inch wire. (d) A silicon-based stopper is linked to the proximal shaft of the sheathless guiding catheter and anchored to a surgical drape using forceps to avoid slippage.

After administration of nitroglycerin oral spray (0.3 mg; Myocor Spray; Toaeiyo, Tokyo, Japan) to patients with ACS, other than those with Killip class IV, the radial artery was punctured 2 cm proximal to the radius styloid process using the Seldinger technique (posterior wall puncture) with 18-Ga puncture needles (Supercath; Medikit, Tokyo, Japan) under local anesthesia. The choice of guiding system (7.5-Fr sheathless guiding catheter or 7-Fr Glidesheath slender sheath/7-Fr guiding catheter combination) was at the operator's discretion. In cases where a 16 cm-long 7-Fr Glidesheath slender sheath was selected, after coronary angiography using a 4-Fr diagnostic catheter with a 7-Fr Glidesheath slender sheath, a 7-Fr Hyperion guiding catheter equipped with an inner dilator (5.5-Fr STA Angiography Catheter; Medikit, Tokyo, Japan) was inserted into the radial artery via the Glidesheath slender. In this study, a 16 cm-long 7-Fr Glidesheath slender was used, while a 10 cm-long one was not available. In all patients who underwent PCI using 7-Fr Glidesheath slender sheaths, 7-Fr Hyperion guiding catheters were used for coronary intubation. In cases where the 7.5-Fr sheathless guiding catheter was selected, a sheath-cum-sheathless technique was used. After coronary angiography using a 4-Fr diagnostic catheter with a 4-Fr conventional sheath, a 4-Fr conventional sheath was subsequently exchanged for a 7.5-Fr sheathless guiding catheter connected to a supplied central dilator over a J-tipped 0.035-inch guidewire and inserted into the radial artery (Figure 3). All PCI procedures were performed after intra-arterial heparin administration (10,000 U). The activated clotting time (ACT) was noted 3 min after the first administration of heparin and every 1 h thereafter. Heparin was administered additionally to maintain an ACT of ≥300 s. End-procedural ACT was immediately measured before removing the guiding catheter at the end of the procedure. After removing the sheathless guiding catheter or the Glidesheath slender sheath, the punctured radial artery was sealed with a compression device (TOMETA KUN; Zeon Medical, Tokyo, Japan) [11] and pressurized up to the reference pressure (defined as ≥20 mmHg over the systolic blood pressure at the time of guiding catheter removal). Thirty minutes after removal, the pressure was dropped by 20 mmHg every 1 h until it reached 20 mmHg. The compression device was removed 8–9 h after the procedure. In all patients, hemostasis was performed according to the aforementioned protocol, which was an occlusive hemostasis technique.

Ultrasound measurements of the radial artery were taken 30 days after the index PCI. A single experienced vascular sonographer, who had no knowledge of the techniques and the devices used for PCI, performed all ultrasonography examinations using Doppler ultrasonography with the Toshiba Aplio XG SSA-790A (Toshiba Medical Systems Corporation, Otawara, Japan) and a PLT-1204AT (2D, 12 MHz) or a PLT-1204BT (2D, 12 MHz) probe or with the Aplio i800 (Canon Medical Systems, Tustin, CA) with an i24LX8 (2D, 24 MHz) probe. The measurements were made 2 cm proximal to the styloid process of the radius. The arterial puncture site and subsequent sheath insertion site were identified by visual confirmation using the skin scar at the sheath insertion site as a landmark. Thus, arterial puncture, sheath insertion, and ultrasound measurement were done at the same site. The radial artery diameter was defined as the distance from the leading edge of the near wall to the leading edge of the far wall of the artery along a line perpendicular to the long axis of the artery.

### 2.3. Definition of Endpoints

The endpoints were RAO at 30 days, severe radial spasm during PCI, access-site major bleeding within 30 days as defined by the Bleeding Academic Research Consortium (BARC) type 3 or 5 criteria [12], coronary ostial dissection by the guiding catheters, procedural success, and clinical outcomes within 30 days. RAO was defined as severely reduced or absent blood flow at the puncture site as revealed by Doppler studies. The Doppler criteria for diagnosing RAO were based on a previous study [13]. If the Doppler measurement could not identify any residual flow, the radial flow was graded as 0. This meant that the radial artery had occluded completely. If it indicated severely reduced antegrade flow in comparison to the contralateral side, the radial flow was graded as 1. This meant that the radial artery was pulseless. In our study, RAO was defined as severely reduced (grade 1) or absent blood flow (grade 0) at the puncture site as revealed by Doppler studies. Severe radial spasm was defined as severe local pain and discomfort during catheter movement that prompted the operator to stop the procedure and crossover to the other route (grade 3) or severe local pain and discomfort associated with catheter trapping (grade 4) [3]. For better objectivity, two experienced research staff members, as independent observers, judged the incidence and severity of radial spasm based on the patients' complaints and medical records on the day of the PCI procedure. Procedural success was defined as the successful completion of transradial PCI that reached a postprocedural thrombolysis in myocardial infarction grade 3 flow and <30% coronary residual stenosis. Thirty-day clinical outcomes included all-cause death, myocardial infarction, stent thrombosis, target-lesion revascularization (TLR), and stroke.

### 2.4. Statistical Analysis

Continuous variables are expressed as median (interquartile range (IQR)), and categorical variables are expressed as frequency (%). Continuous variables were compared using the Mann–Whitney *U* test, while categorical variables were compared using Fisher's exact test. For alleviating potential selection bias between the two groups, a propensity score was estimated using a multivariate logistic regression model. The co-variables included in the model were selected based on the results of the univariate analysis/clinical perspective including age, sex, body mass index, diabetes mellitus, hypertension, dyslipidemia, current smoking, peripheral artery disease, history of previous bleeding requiring hospitalization or transfusion, history of stroke and myocardial infarction, previous PCI, previous coronary artery bypass grafting, use of oral anticoagulants, Killip class on admission, estimated glomerular filtration rate, arterial access site (left or right radial artery approach), and the number of previous ipsilateral transradial coronary angiography or intervention attempts [2, 14]. Notably, these co-variables only included those that would be known at the time of the point of catheter selection. The C-statistic for the propensity score model was 0.62, and 1 : 1 matching on the propensity score was performed using nearest neighbor matching with a maximum caliper of 0.05 of the propensity score. In both groups, clinical outcomes within 30 days were compared using the Cox proportional hazard models and Kaplan–Meier method. A two-tailed *p* value of <0.05 was considered statistically significant. All statistical analyses were performed using JMP software (version 14, SAS Institute Inc., Cary, NC, USA). The initial sample size calculation was performed to detect a 4% difference in the incidences of RAO (Sheathless group: 0.8%; Glidesheath group: 4.8%) with a power of 80% and type I error of 5%. A sample size of 632 patients (316 in each group) was required [5, 6]. At the time of planning the study, the data on the incidence of RAO with 7.5-Fr sheathless guiding catheters in patients with ACS were not available. We, therefore, presumed that the incidence of RAO would be close to that of the 6-Fr Glidesheath slender, which has an outer diameter of 2.45 mm, as the outer diameter of the 7.5-Fr sheathless guiding catheter is similar, at 2.49 mm.

### 2.5. Ethics Approval

The study was approved by the Institutional Research Committee of the Sendai Kousei Hospital (approval no: 1-97). All procedures performed in this study were in accordance with the ethical standards of the Institutional Research Committee and the 1964 Helsinki Declaration and its later amendments or comparable ethical standards.

## 3. Results

The study sample included 1108 patients who underwent transradial PCI for ACS (Glidesheath group, *n* = 397; Sheathless group, *n* = 711). Following propensity score matching, 728 patients treated with PCI using a 7-Fr Glidesheath slender sheath/7-Fr guiding catheter combination or a 7.5-Fr sheathless guiding catheter were included in the matched population (Glidesheath group, *n* = 364; Sheathless group, *n* = 364). Key baseline clinical and procedural characteristics of the study population are summarized in Tables 1 and 2. In the unmatched patients, the Glidesheath group exhibited a significantly higher percentage of patients with current smoking, previous PCI, left circumflex artery lesions, and heavily calcified lesions and significantly less previous ipsilateral transradial angiography attempts than the Sheathless group. After propensity score matching, the baseline clinical and procedural characteristics were well balanced between the groups, except for the frequency of left circumflex artery lesions, the guiding catheter types used to perform PCI for left anterior descending or diagonal artery, and the rate of thrombus aspiration.

The procedural outcomes are shown in Table 3. In the matched patients, the procedural success rates were high for the Glidesheath and Sheathless groups, with no significant differences between them. The total fluoro time was significantly longer in the Glidesheath group than in the Sheathless group (median [IQR], Glidesheath; 22.3 min [15.3–31.5] vs. Sheathless; 18.7 min [13.8–29.5], *p*=0.002). The median number of catheters used per procedure was one in each group.

The periprocedural access-site complications are presented in Table 4. In the matched patients, the Glidesheath group was significantly less likely to develop ultrasound-diagnosed RAO at 30 days compared to the Sheathless group (Glidesheath: 1.4% vs. Sheathless: 4.1%, odds ratio (OR) 0.33, 95% confidence interval (CI) 0.12–0.91; *p*=0.039), whereas the incidences of severe radial spasm and access-site major bleeding defined by BARC type 3 or 5 criteria were not significantly different between the two groups. Multivariate logistic regression analysis of the matched patients to determine predictors of RAO at 30 days revealed that the choice for Glidesheath was significantly associated with less RAO at 30 days (OR 0.32, 95% CI 0.11–0.93; *p*=0.036). Conversely, end-procedural ACT and total fluoro time were not significantly associated with RAO at 30 days (Table 5). The 30-day clinical outcomes are presented in Table 6. There were no significant between-group differences in the incidences of all-cause death, myocardial infarction, stent thrombosis, and TLR. Kaplan–Meier curve analysis results for individual components of 30-day clinical outcomes according to the type of guiding system are shown in Supplementary Figures 1–5.

## 4. Discussion

To our knowledge, the present study is one of the first studies to compare the 7-Fr Glidesheath slender sheath/7-Fr guiding catheter combination with a 7.5-Fr sheathless Eaucath guiding catheter in a large cohort of patients undergoing transradial PCI for ACS. One of the strengths of the study is the assessment of RAO incidence using ultrasound examination 30 days after the procedure. The main findings were (i) incidence of RAO at 30 days was significantly less frequent when a Glidesheath slender/guiding catheter combination was used and (ii) overall incidences of access-site complications were low in the Glidesheath and Sheathless groups, with no significant differences between them except for RAO.

First, in the matched comparison, compared with that observed with a sheathless guiding catheter, the incidence of ultrasound-diagnosed postprocedural RAO at 30 days was significantly less frequent when a Glidesheath slender sheath/guiding catheter combination was used, although the outer diameter of the 7-Fr Glidesheath slender is larger than that of the 7.5-Fr sheathless guiding catheter by 0.30 mm and a larger-bore device causes more RAO [2]. The reason why we found the reverse might be due to more injury to radial artery by exchanging the 4-Fr conventional sheath for a sheathless guiding catheter. That is, exchanging a 4-Fr sheath for a sheathless guiding catheter over a 0.035-inch guidewire may cause more extensive dissection of radial artery, resulting in more RAO incidence. This speculation is supported by a recent study using optical coherence tomography imaging, revealing that sheathless guiding system was not related to reduced radial injury and caused more medial dissection when compared to conventional sheath/guiding system [15]. In our opinion, the different nature of radial artery injury at the puncture site might have caused the significantly different incidences of RAO between the two groups: a 7-Fr Glidesheath slender with a dilator directly tracking on a guidewire after radial puncture versus graded injury by a 4-Fr sheath, followed by a 7.5-Fr sheathless guiding catheter on the dilator. Studies have shown that a larger-bore device causes more RAO, and the outer diameter of the 7-Fr Glidesheath slender is larger than that of the 7.5-Fr sheathless guiding catheter by 0.30 mm. However, more local damage to the puncture site by exchanging the 4-Fr conventional sheath for a sheathless guiding catheter may explain why we found the reverse in our study. In contrast, the role of the length of the sheathless guiding system (outer diameter 2.49 mm all the way to the coronary artery) as compared to the 16 cm-long Glidesheath slender system (outer diameter 2.79 mm for the length of the sheath) may have had little impact on the incidences of RAO because when a 16 cm-long 7-Fr Glidesheath slender sheath is introduced into the radial artery, the tip of the sheath will reach the very proximal radial artery or the brachial artery. As a result, both the sheathless guiding catheter and Glidesheath slender sheath are placed along the entire length of the radial artery. Both would, therefore, damage the entire radial artery similarly. Notably, unlike a sheathless guiding catheter, a Glidesheath slender sheath does not require exchange of sheaths in performing PCI, which may contribute to avoiding extensive radial injury at the sheath insertion site. In case of exchanging the guiding catheter for a larger/smaller size, more injury to radial artery is not expected in the Glidesheath group; the guiding catheters pass inside the 16 cm-long Glidesheath slender sheath and do not cause more damage to radial artery at the sheath insertion point. Therefore, the number of catheters used per procedure may not be related to the radial injury in the Glidesheath group and we did not include it in the multivariate analysis to determine predictors of RAO. Nevertheless, the two slender devices revealed low incidences of postprocedural RAO in our study (1.4% for Glidesheath and 4.1% for Sheathless). Notably, with both the slender devices, the incidences of RAO were lower than or almost equal to those of conventional 6-Fr sheaths/guiding catheters. These results were reasonable, considering that the outer diameters of these two devices are almost equal or smaller than those of conventional 6-Fr sheaths. By comparison, the incidences of RAO of conventional 6-Fr sheaths are reportedly 3.5%–15.2% at ≥2 days after PCI [2]. Therefore, our data suggest that using both the 7-Fr Glidesheath slender sheath and 7.5-Fr sheathless guiding catheter have equal or lower risk of RAO compared to conventional 6-Fr sheaths. Our study population was limited to Japanese patients; therefore, we do not know whether these results can be generalized to other populations. However, these slender devices may be better in European or American patients because their radial artery size is usually larger than that of Japanese patients [16], resulting in less postprocedural RAO. Notably, in the Glidesheath group, we only documented six cases of RAO in the total population and five in the propensity-matched population, yielding the incidences of RAO of 1.5% and 1.4%, respectively, which are lower than the incidence previously reported for a 7-Fr Glidesheath slender sheath (4.8%) [5]. Factors like intraprocedural anticoagulation of maintaining an ACT of 300 s or longer might have contributed to this low incidence of RAO.

Second, the overall incidences of access-site complications were low in the Glidesheath and Sheathless groups with no significant differences between them except for RAO; the incidence of severe radial spasm during PCI was low, but not significantly different between both them in the present study. This is not consistent with our previous study on elective transradial PCI using a 6.5-Fr sheathless guiding catheter vs. a 6-Fr Glidesheath slender [9]. The advanced hydrophilic surface coating, which is common to both the devices, might have made the detection of slight difference difficult. Moreover, the assessment of radial spasm based on the subjective feelings of patients and subjective observations by operators may also have led to inconsistent results. Access-site major bleeding events were not significantly different between the two groups. This is contradictory to a previous meta-analysis that demonstrated that a 5-Fr system in transradial PCI significantly reduces bleeding frequency compared to a 6-Fr system [17]; the difference in outer sheath size between the 5-Fr and 6-Fr sheaths was approximately 0.4 mm. This suggested that the difference in outer sheath size between 7-Fr Glidesheath slender and 7.5-Fr sheathless guiding catheter (0.3 mm) was too small to cause a significant difference in the incidence of access-site major bleeding events between the two groups.

Finally, the rates of catheter-induced coronary dissection were not significantly different between the two groups. In our study (*n* = 1108), coronary ostial dissection occurred in 13 patients. Of note, 9 patients experienced right coronary artery (RCA) ostial dissection (2.8% (6/217) for the Sheathless group and 2.2% (3/139) for the Glidesheath group, *p*=0.75, respectively). One of the greatest concerns with the use of sheathless guiding catheters in PCI is the risk of catheter-induced coronary dissection due to the greater tip stiffness of their double-braiding design. In our opinion, RCA dissections may have been directly related to the lesion characteristics and not the choice of the sheathless guiding catheter because in our study, most dissections occurred in RCAs with moderate ostial stenosis. Therefore, in patients with moderate ostial stenosis of the RCA, caution should be exercised during positioning and coaxial alignment of the guiding catheters with the artery, irrespective of the catheter type.

## 5. Study Limitations

There are several limitations to our study. First, this was a retrospective observational single-center study, and therefore, unknown associated factors in access-site complications may have been unequally distributed between the groups. Second, the baseline clinical and the procedural characteristics of the study population may not have been sufficiently adjusted despite propensity score matching. As a result, more difficult cases might have been included in the Glidesheath group, which would explain the significantly longer fluoroscopy time. The shorter fluoroscopy time in the Sheathless group, interpreted differently, may indicate higher operator confidence, preference, and familiarity with the sheathless guiding system because the co-variables regarding propensity score matching did not include operator-related factors. Third, a randomized controlled trial would be needed to further validate our findings. Fourth, data pertaining to the pre-PCI Doppler evaluation were not obtained. Indeed, assessing the radial artery diameter 30 days after PCI would have likely only introduced a slight error in the measurement of radial artery size because the lumen of the radial artery at the puncture site remained unchanged during follow-up, except for the distal part of the puncture site [18]. The variables that might have influenced the size of the preprocedure radial artery, including sex and body mass index [16], were well balanced after propensity score matching. However, in reality, we do not know whether or not the preprocedure radial artery diameter of the Sheathless group was the same as that of the Glidesheath group. Fifth, radial artery diameter is influenced by smoking [19]. We have no data on how many patients had quit smoking at 30-day follow-up; however, many of them would have quit smoking because we advised all our patients to quit smoking during hospitalization. Consequently, the preprocedure radial artery diameter could have been slightly different from the postprocedure diameter. Sixth, despite statistical significance, the wide confidence interval of the RAO result indicates that information needs to be collected from a larger sample of patients with ACS to strengthen the association. Lastly, morphology of acute radial artery injuries after PCI was not assessed by optical coherence tomography, and therefore, radial injury by sheath exchange could not be completely identified as the immediate cause of more frequency of RAO in the Sheathless group.

## 6. Conclusions

We conducted a propensity score-based comparison of 7.5-Fr sheathless guiding catheters vs. 7-Fr Glidesheath slender sheaths/7-Fr guiding catheter combination in PCI for ACS. We found that 7-Fr Glidesheath slender sheaths/7-Fr guiding catheter combination significantly reduced RAO, while maintaining a low incidence of severe radial spasm or access-site major bleeding and keeping an acceptable procedural success rate, with no significant differences between the groups. Our data suggest a greater advantage of Glidesheath slender sheaths over sheathless guiding catheters for decreased risk of RAO and inspire the expansion of the “slender sheath-first” approach in transradial PCI for ACS.

## Figures and Tables

**Table 1:** Baseline clinical characteristics of the study population.

Variables	Total population			Propensity-matched population		
	Glidesheath *n* = 397	Sheathless *n* = 711	*p*	Glidesheath *n* = 364	Sheathless *n* = 364	*p*
Age, y	68.0 (58.0–77.0)	68.0 (58.0–77.0)	0.89	67.5 (57.3–76.0)	67.0 (57.0–75.0)	0.69
Sex (male), *n* (%)	318 (80.1)	554 (77.9)	0.44	292 (80.2)	289 (79.4)	0.85
Height, cm	165.0 (160.0–170.0)	164.5 (158.0–170.0)	0.068	165.7 (160.0–170.0)	165.0 (158.0–170.0)	0.23
Weight, kg	67.0 (58.0–76.0)	65.0 (57.0–74.0)	0.12	67.6 (58.0–77.0)	66.8 (58.0–75.5)	0.54
Body mass index, kg/m^2^	24.6 (22.3–27.2)	24.3 (22.4–26.8)	0.51	24.6 (22.4–27.3)	24.6 (22.6–27.4)	0.64
Diabetes mellitus, *n* (%)	167 (42.1)	259 (36.4)	0.071	150 (41.2)	157 (43.1)	0.65
Hypertension, *n* (%)	315 (79.4)	557 (78.3)	0.76	286 (78.6)	282 (77.5)	0.79
Dyslipidemia, *n* (%)	270 (68.0)	475 (66.8)	0.69	243 (66.8)	247 (67.9)	0.81
Current smoker, *n* (%)	171 (43.1)	240 (33.8)	0.002^*∗*^	151 (41.5)	152 (41.8)	1.00
Peripheral artery disease, *n* (%)	11 (2.8)	24 (3.4)	0.72	9 (2.5)	12 (3.3)	0.66
Previous history of bleeding, *n* (%)	32 (8.1)	59 (8.3)	1.0	31 (8.5)	32 (8.8)	1.00
Previous history of heart failure, *n* (%)	25 (6.3)	36 (5.1)	0.41	18 (5.0)	21 (5.8)	0.74
Previous stroke, *n* (%)	21 (5.3)	45 (6.3)	0.51	20 (5.5)	23 (6.3)	0.75
Previous MI, *n* (%)	27 (6.8)	70 (9.9)	0.096	25 (6.9)	28 (7.7)	0.78
Previous PCI, *n* (%)	52 (13.1)	133 (18.7)	0.019^*∗*^	46 (12.6)	46 (12.6)	1.00
Previous CABG, *n* (%)	3 (0.8)	3 (0.4)	0.67	3 (0.8)	2 (0.6)	1.00
Oral anticoagulants, *n* (%)	39 (9.9)	75 (10.6)	0.76	34 (9.3)	35 (9.6)	1.00
Killip IV on admission, *n* (%)	13 (3.3)	14 (2.0)	0.22	9 (2.5)	10 (2.8)	1.00
eGFR, ml/min/1.73 m^2^	74.6 (59.3–91.0)	75.1 (60.6–86.9)	0.87	75.4 (60.1–91.5)	77.3 (62.8–88.8)	0.72

Data are presented as median (interquartile range) or *n* (%), unless otherwise indicated. “Glidesheath” denotes 7-Fr Glidesheath slender/7-Fr guiding catheter combination group, and “Sheathless” denotes 7.5-Fr sheathless guiding catheter group. The asterisk denotes a statistically significant difference between the two groups. CABG, coronary artery bypass grafting; eGFR, estimated glomerular filtration rate; MI, myocardial infarction; PCI, percutaneous coronary intervention.

**Table 2:** Procedural characteristics of the study population.

Variables	Total population			Propensity-matched population		
	Glidesheath *N* = 397	Sheathless *N* = 711	*p*	Glidesheath *N* = 364	Sheathless *N* = 364	*p*
LAD/diagonal, *n* (%)	196 (49.4)	318 (44.7)	0.15	179 (49.2)	170 (46.7)	0.55
LCX/marginal, *n* (%)	37 (9.3)	144 (20.3)	<0.001^*∗*^	33 (9.1)	72 (19.8)	<0.001^*∗*^
RCA, *n* (%)	139 (35.0)	217 (30.5)	0.14	132 (36.3)	109 (30.0)	0.08
LMCA, *n* (%)	25 (6.3)	32 (4.5)	0.20	20 (5.5)	13 (3.6)	0.29
Guiding catheter type						
LAD/diagonal (JL/EBU/others), *n* (%)	143 (73.0)/51 (26.0)/2 (1.0)	263 (82.7)/54 (17.0)/1 (0.3)	0.025^*∗*^	132 (73.7)/46 (25.7)/1 (0.6)	144 (84.7)/26 (15.3)/0 (0)	0.033^*∗*^
LCX/marginal (JL/EBU/others), *n* (%)	13 (35.1)/23 (62.2)/1 (2.7)	79 (54.9)/65 (45.1)/0 (0)	0.019^*∗*^	12 (36.4)/20 (60.6)/1 (3.0)	42 (58.3)/30 (41.7)/0 (0)	0.050
RCA (JR/AL/others), *n* (%)	101 (72.7)/30 (21.6)/8 (5.7)	157 (72.4)/58 (26.7)/2 (0.9)	0.018^*∗*^	97 (73.5)/28 (21.2)/7 (5.3)	81 (74.3)/26 (23.9)/2 (1.8)	0.22
LMCA (JL/EBU/others), *n* (%)	15 (60.0)/9 (36.0)/1 (4.0)	28 (87.5)/3 (9.4)/1 (3.1)	0.0046^*∗*^	14 (70.0)/6 (30.0)/0 (0)	12 (92.3)/1 (7.7)/0 (0)	0.12
True bifurcation lesion, *n* (%)	67 (16.9)	99 (13.9)	0.19	58 (15.9)	51 (14.0)	0.53
In-stent restenosis/occlusion, *n* (%)	26 (6.6)	39 (5.5)	0.58	24 (6.6)	18 (5.0)	0.43
Diffuse lesion, *n* (%)	264 (66.5)	434 (61.0)	0.080	246 (67.6)	228 (62.6)	0.19
Heavily calcified lesion, *n* (%)	13 (3.3)	10 (1.4)	0.047^*∗*^	12 (3.3)	5 (1.4)	0.14
Thrombus aspiration, *n* (%)	218 (54.9)	253 (35.6)	<0.001^*∗*^	203 (55.8)	135 (37.1)	<0.001^*∗*^
Rotablation, *n* (%)	8 (2.0)	6 (0.8)	0.16	7 (1.9)	2 (0.6)	0.18
Arterial access site						
Left radial, *n* (%)	373 (94.0)	679 (95.5)	0.26	344 (94.5)	343 (94.2)	1.00
No. of previous iTRA attempts, median (min–max)	0 (0–9)	0 (0–11)	0.002^*∗*^	0 (0–9)	0 (0–11)	0.17
No. of previous iTRI attempts, median (min–max)	0 (0–7)	0 (0–7)	0.059	0 (0–7)	0 (0–7)	0.36

Data are presented as median (interquartile range) or *n* (%), unless otherwise indicated. “Glidesheath” denotes 7-Fr Glidesheath slender/7-Fr guiding catheter combination group, and “Sheathless” denotes 7.5-Fr sheathless guiding catheter group. The asterisk denotes a statistically significant difference between the two groups. AL, Amplatz type; EBU, extra backup type; iTRA, ipsilateral transradial coronary angiography; iTRI, ipsilateral transradial coronary intervention; JL, Judkins Left type; JR, Judkins Right type; LAD, left anterior descending coronary artery; LCX, left circumflex coronary artery; LMCA, left main coronary artery; RCA, right coronary artery.

**Table 3:** Procedural outcomes.

Variables	Total population				Propensity-matched population			
	Glidesheath *N* = 397	Sheathless *N* = 711	OR (95% CI)	*p*	Glidesheath *N* = 364	Sheathless *N* = 364	OR (95% CI)	*p*
Procedural success, *n* (%)	392 (98.7)	702 (98.7)	1.00 (0.99–1.01)	1.0	359 (98.6)	361 (99.2)	0.99 (0.98–1.01)	0.73
Coronary ostial dissection, *n* (%)	3 (0.8)	10 (1.4)	1.86 (0.52–6.72)	0.40	3 (0.8)	6 (1.7)	0.50 (0.13–1.98)	0.51
Access-site crossover from radial to femoral, *n* (%)	1 (0.3)	0 (0)	n/a	0.36	1 (0.3)	0 (0)	n/a	1.00
Total fluoroscopy time, min	22.4 (15.3–31.5)	18.2 (13.9–28.0)		<0.001^*∗*^	22.3 (15.3–31.5)	18.7 (13.8–29.5)		0.002^*∗*^
Contrast used, ml	128 (100–160)	127 (103–160)		0.67	128 (100–160)	133 (105–165)		0.11
No. of catheters used, median (min–max)	1 (1–5)	1 (1–4)		<0.001^*∗*^	1 (1–5)	1 (1–4)		0.016^*∗*^

Data are presented as median (interquartile range) or *n* (%), unless otherwise indicated. “Glidesheath” denotes 7-Fr Glidesheath slender/7-Fr guiding catheter combination group, and “Sheathless” denotes 7.5-Fr sheathless guiding catheter group. The asterisk denotes a statistically significant difference between the two groups. CI, confidence interval; n/a, not applicable; OR, odds ratio.

**Table 4:** Periprocedural access-site complications.

Variables	Total population				Propensity-matched population			
	Glidesheath *N* = 397	Sheathless *N* = 711	OR (95% CI)	*p*	Glidesheath *N* = 364	Sheathless *N* = 364	OR (95% CI)	*p*
RAD at 30 days, mm	2.0 (1.8–2.3)	2.1 (1.8–2.4)		0.15	2.0 (1.8–2.3)	2.1 (1.8–2.4)		0.06
End-procedural ACT, s	273 (231–312)	301 (250–376)		<0.001^*∗*^	273 (229–310)	289 (250–372)		<0.001^*∗*^
RAO at 30 days, *n* (%)	6 (1.5)	25 (3.5)	0.43 (0.18–1.04)	0.058	5 (1.4)	15 (4.1)	0.33 (0.12–0.91)	0.039^*∗*^
Severe radial spasm, *n* (%)	9 (2.3)	11 (1.6)	1.46 (0.61–3.49)	0.48	5 (1.4)	7 (1.9)	0.71 (0.23–2.22)	0.58
*Access-site major bleeding within* 30 *days*								
BARC type 3 or 5, *n* (%)	5 (1.3)	9 (1.3)	0.99 (0.34–2.95)	1.00	5 (1.4)	6 (1.6)	0.83 (0.26–2.71)	1.00
BARC type 3, *n* (%)	5 (1.3)	9 (1.3)	0.99 (0.34–2.95)	1.00	5 (1.4)	6 (1.6)	0.83 (0.26–2.71)	1.00
BARC type 5, *n* (%)	0 (0)	0 (0)	n/a	n/a	0 (0)	0 (0)	n/a	n/a

Data are presented as median (interquartile range) or *n* (%), unless otherwise indicated. “Glidesheath” denotes 7-Fr Glidesheath slender/7-Fr guiding catheter combination group, and “Sheathless” denotes 7.5-Fr sheathless guiding catheter group. The asterisk denotes a statistically significant difference between the two groups. ACT; activated clotting time; BARC, Bleeding Academic Research Consortium; CI, confidence interval; n/a, not applicable; OR, odds ratio; RAD, radial artery diameter; RAO, radial artery occlusion.

**Table 5:** Multivariate logistic regression analysis to determine predictors of radial artery occlusion at 30 days.

Variables	Adjusted OR (95% CI)	*p*
Treatment modality (Glidesheath/Sheathless)	0.32 (0.11–0.93)	0.036^*∗*^
End-procedural ACT, per s	1.00 (0.99–1.00)	0.30
Total fluoroscopy time, per min	1.02 (1.00–1.04)	0.055

“Glidesheath” denotes 7-Fr Glidesheath slender/7-Fr guiding catheter combination group, and “Sheathless” denotes 7.5-Fr sheathless guiding catheter group. Of note, the number of catheters used per procedure was not included in the multivariate analysis. ACT, activated clotting time; CI, confidence interval; OR, odds ratio.

**Table 6:** Thirty-day clinical outcomes by Cox proportional hazard ratio model analysis.

Variables	Cumulative events at 30 days (%)		Hazard ratio (95% CI)	*p*
	Glidesheath	Sheathless		
*Total population*				
All-cause death	4 (1.1)	1 (0.2)	7.30 (1.08–142.74)	0.075
Myocardial infarction	0 (0)	2 (0.3)	0.83 (0.04–8.67)	0.88
Stent thrombosis	4 (1.0)	1 (0.1)	8.94 (1.44–171.24)	0.046^*∗*^
TLR	0 (0)	1 (0.1)	1.68 (0.07–42.61)	0.71
Stroke	2 (0.5)	1 (0.1)	5.12 (0.65–103.56)	0.16

*Propensity-matched population*				
All-cause death	4 (1.2)	1 (0.3)	4.06 (0.60–79.31)	0.21
Myocardial infarction	0 (0)	1 (0.3)	0.87 (0.03–22.09)	0.92
Stent thrombosis	3 (0.8)	1 (0.3)	3.98 (0.59–77.80)	0.22
TLR	0 (0)	1 (0.3)	0.99 (0.86–1.15)	0.96
Stroke	2 (0.6)	0 (0)	n/a	n/a

“Glidesheath” denotes the 7-Fr Glidesheath slender/7-Fr guiding catheter combination group and “Sheathless” denotes the 7.5-Fr sheathless guiding catheter group. The asterisk denotes a statistically significant difference between the two groups. CI, confidence interval; n/a, not applicable; TLR, target-lesion revascularization.

## Data Availability

The data used to support the findings of this study are available from the corresponding author upon request.

## Supplementary Materials

Supplementary Materials Supplementary Figure 1: Kaplan–Meier curve analysis of all-cause death at 30 days according to the type of guiding system. “Glidesheath” denotes 7-Fr Glidesheath slender/7-Fr guiding catheter combination group and “Sheathless” denotes 7.5-Fr sheathless guiding catheter group; PCI, percutaneous coronary intervention. Supplementary Figure 2: Kaplan–Meier curve analysis of myocardial infarction at 30 days according to the type of guiding system. “Glidesheath” denotes 7-Fr Glidesheath slender/7-Fr guiding catheter combination group and “Sheathless” denotes 7.5-Fr sheathless guiding catheter group. Supplementary Figure 3: Kaplan–Meier curve analysis of stent thrombosis at 30 days according to the type of guiding system. “Glidesheath” denotes 7-Fr Glidesheath slender/7-Fr guiding catheter combination group and “Sheathless” denotes 7.5-Fr sheathless guiding catheter group. Supplementary Figure 4: Kaplan–Meier curve analysis of TLR at 30 days according to the type of guiding system. “Glidesheath” denotes 7-Fr Glidesheath slender/7-Fr guiding catheter combination group and “Sheathless” denotes 7.5-Fr sheathless guiding catheter group; TLR, target-lesion revascularization. Supplementary Figure 5: Kaplan–Meier curve analysis of stroke at 30 days according to the type of guiding system. “Glidesheath” denotes 7-Fr Glidesheath slender/7-Fr guiding catheter combination group and “Sheathless” denotes 7.5-Fr sheathless guiding catheter group. Supplementary Figure 6: graphical abstract.
