# Erratum to “Slender Sheath/Guiding Catheter Combination vs. Sheathless Guiding Catheter for Acute Coronary Syndrome: A Propensity-Matched Analysis of the Two Devices”

**DOI:** 10.1155/2020/1303764

**Published:** 2020-10-22

**Authors:** Tsuyoshi Isawa, Kazunori Horie, Taku Honda, Masataka Taguri, Norio Tada

**Affiliations:** ^1^Department of Cardiology, Sendai Kousei Hospital, Sendai, Japan; ^2^Department of Data Science, Yokohama City University School of Data Science, Yokohama, Japan

In the article titled “Slender Sheath/Guiding Catheter Combination vs. Sheathless Guiding Catheterfor Acute Coronary Syndrome: A Propensity-Matched Analysis of the Two Devices” [1], there were errors in Tables 2, 3, and 4. In each of these tables the *n* value was incorrectly given as *n* = 397 for the propensity-matched population in both the Glidesheath and Sheathless columns. The correct value is *n* = 364. This mistake occurred during the production of the article, and the publisher apologises for this error.

The corrected tables are shown below and are listed as Tables 2–4, respectively.

## Figures and Tables

**Table 2:** Procedural characteristics of the study population.

Variables	Total population			Propensity-matched population		
	Glidesheath *n* = 397	Sheathless *n* = 711	*P*	Glidesheath *n* = 364	Sheathless *n* = 364	*P*
LAD/diagonal, *n* (%)	196 (49.4)	318 (44.7)	0.15	179 (49.2)	170 (46.7)	0.55
LCX/marginal, *n* (%)	37 (9.3)	144 (20.3)	<0.001	33 (9.1)	72 (19.8)	<0.001
RCA, *n* (%)	139 (35.0)	217 (30.5)	0.14	132 (36.3)	109 (30.0)	0.08
LMCA, *n* (%)	25 (6.3)	32 (4.5)	0.20	20 (5.5)	13 (3.6)	0.29

*Guiding catheter type*						
LAD/diagonal (JL/EBU/others), *n* (%)	143 (73.0)/51 (26.0)/2(1.0)	263 (82.7)/54 (17.0)/1 (0.3)	0.025	132 (73.7)/46 (25.7)/1 (0.6)	144 (84.7)/26 (15.3)/0(0)	0.033
LCX/marginal (JL/EBU/others), *n* (%)	13 (35.1)/23 (62.2)/1 (2.7)	79 (54.9)/65 (45.1)/0 (0)	0.019	12 (36.4)/20 (60.6)/1 (3.0)	42 (58.3)/30 (41.7)/0(0)	0.050
RCA (JR/AL/others), *n* (%)	101 (72.7)/30 (21.6)/8 (5.7)	157 (72.4)/58 (26.7)/2 (0.9)	0.018	97 (73.5)/28 (21.2)/7 (5.3)	81 (74.3)/26 (23.9)/2(1.8)	0.22
LMCA (JL/EBU/others), *n* (%)	15 (60.0)/9 (36.0)/1 (4.0)	28 (87.5)/3 (9.4)/1 (3.1)	0.0046	14 (70.0)/6 (30.0)/0 (0)	12 (92.3)/1 (7.7)/0 (0)	0.12
True bifurcation lesion, *n* (%)	67 (16.9)	99 (13.9)	0.19	58 (15.9)	51 (14.0)	0.53
In-stent restenosis/occlusion, *n* (%)	26 (6.6)	39 (5.5)	0.58	24 (6.6)	18 (5.0)	0.43
Diffuse lesion, *n* (%)	264 (66.5)	434 (61.0)	0.080	246 (67.6)	228 (62.6)	0.19
Heavily calcified lesion, *n* (%)	13 (3.3)	10 (1.4)	0.047	12 (3.3)	5 (1.4)	0.14
Thrombus aspiration, *n* (%)	218 (54.9)	253 (35.6)	<0.001	203 (55.8)	135 (37.1)	<0.001
Rotablation, *n* (%)	8 (2.0)	6 (0.8)	0.16	7 (1.9)	2 (0.6)	0.18

*Arterial access site*						
Left radial, *n* (%)	373 (94.0)	679 (95.5)	0.26	344 (94.5)	343 (94.2)	1.00
No. of previous iTRA attempts, median (min–max)	0 (0–9)	0 (0–11)	0.002	0 (0–9)	0 (0–11)	0.17
No. of previous iTRI attempts, median (min–max)	0 (0–7)	0 (0–7)	0.059	0 (0–7)	0 (0–7)	0.36

Data are presented as median (interquartile range) or *n* (%), unless otherwise indicated. “Glidesheath” denotes 7-Fr Glidesheath slender/7-Fr guiding catheter combination group, and “Sheathless” denotes 7.5-Fr sheathless guiding catheter group. The asterisk denotes a statistically significant difference between the two groups. AL, Amplatz type; EBU, extra backup type; iTRA, ipsilateral transradial coronary angiography; iTRI, ipsilateral transradial coronary intervention; JL, Judkins Left type; JR, Judkins Right type; LAD, left anterior descending coronary artery; LCX, left circumflex coronary artery; LMCA, left main coronary artery; RCA, right coronary artery.

**Table 3:** Procedural outcomes.

Variables	Total population				Propensity-matched population			
	Glidesheath *n* = 397	Sheathless *n* = 711	OR (95% CI)	*P*	Glidesheath *n* = 364	Sheathless *n* = 364	OR (95% CI)	*P*
Procedural success, *n* (%)	392 (98.7)	702 (98.7)	1.00 (0.99–1.01)	1.0	359 (98.6)	361 (99.2)	0.99 (0.98–1.01)	0.73
Coronary ostial dissection, *n* (%)	3 (0.8)	10 (1.4)	1.86 (0.52–6.72)	0.40	3 (0.8)	6 (1.7)	0.50 (0.13–1.98)	0.51
Access-site crossover from radial to femoral, *n* (%)	1 (0.3)	0 (0)	n/a	0.36	1 (0.3)	0 (0)	n/a	1.00
Total fluoroscopy time, min	22.4 (15.3–31.5)	18.2 (13.9–28.0)		<0.001	22.3 (15.3–31.5)	18.7 (13.8–29.5)		0.002
Contrast used, ml	128 (100–160)	127 (103–160)		0.67	128 (100–160)	133 (105–165)		0.11
No. of catheters used, median (min–max)	1 (1–5)	1 (1–4)		<0.001	1 (1–5)	1 (1–4)		0.016

Data are presented as median (interquartile range) or *n* (%), unless otherwise indicated. “Glidesheath” denotes 7-Fr Glidesheath slender/7-Fr guiding catheter combination group, and “Sheathless” denotes 7.5-Fr sheathless guiding catheter group. The asterisk denotes a statistically significant difference between the two groups. CI, confidence interval; n/a, not applicable; OR, odds ratio.

**Table 4:** Periprocedural access-site complications.

Variables	Total population				Propensity-matched population			
	Glidesheath *n* = 397	Sheathless *n* = 711	OR (95% CI)	*P*	Glidesheath *n* = 364	Sheathless *n* = 364	OR (95% CI)	*P*
RAD at 30 days, mm	2.0 (1.8–2.3)	2.1 (1.8–2.4)		0.15	2.0 (1.8–2.3)	2.1 (1.8–2.4)		0.06

End-procedural ACT, s	273 (231–312)	301 (250–376)		<0.001	273 (229–310)	289 (250–372)		<0.001

RAO at 30 days, *n* (%)	6 (1.5)	25 (3.5)	0.43 (0.18–1.04)	0.058	5 (1.4)	15 (4.1)	0.33 (0.12–0.91)	0.039
Severe radial spasm, *n* (%)	9 (2.3)	11 (1.6)	1.46 (0.61–3.49)	0.48	5 (1.4)	7 (1.9)	0.71 (0.23–2.22)	0.58

*Access-site major bleeding within 30 days*								
BARC type 3 or 5, *n* (%)	5 (1.3)	9 (1.3)	0.99 (0.34–2.95)	1.00	5 (1.4)	6 (1.6)	0.83 (0.26–2.71)	1.00

BARC type 3, *n* (%)	5 (1.3)	9 (1.3)	0.99 (0.34–2.95)	1.00	5 (1.4)	6 (1.6)	0.83 (0.26–2.71)	1.00

BARC type 5, *n* (%)	0 (0)	0 (0)	n/a	n/a	0 (0)	0 (0)	n/a	n/a

Data are presented as median (interquartile range) or *n* (%), unless otherwise indicated. “Glidesheath” denotes 7-Fr Glidesheath slender/7-Fr guiding catheter combination group, and “Sheathless” denotes 7.5-Fr sheathless guiding catheter group. The asterisk denotes a statistically significant difference between the two groups. ACT; activated clotting time; BARC, bleeding academic research consortium; CI, confidence interval; n/a, not applicable; OR, odds ratio; RAD, radial artery diameter; RAO, radial artery occlusion.
